# ‘Holding the line’: a qualitative study of the role of evidence in early phase decision-making in the reconfiguration of stroke services in London

**DOI:** 10.1186/s12961-017-0207-7

**Published:** 2017-06-09

**Authors:** Alec Fraser, Juan I. Baeza, Annette Boaz

**Affiliations:** 1 Department of Health Services Research & Policy, London School of Hygiene and Tropical Medicine, 15-17 Tavistock Place, London, WC1H 9SH United Kingdom; 2 School of Management and Business, King’s College London, 150 Stamford Street, London, SE19NH United Kingdom; 3 Faculty of Health, Social Care and Education, St. George’s, University of London & Kingston University, Grosvenor Wing, Cranmer Terrace, London, SW17 ORE United Kingdom

**Keywords:** England, NHS, Reconfiguration, Healthcare policy, Power, Evidence, Stroke

## Abstract

**Background:**

Health service reconfigurations are of international interest but remain poorly understood. This article focuses on the use of evidence by senior managerial decision-makers involved in the reconfiguration of stroke services in London 2008–2012. Recent work comparing stroke service reconfiguration in London and Manchester emphasises the ability of senior managerial decision-makers in London to ‘hold the line’ in the crucial early phases of the stroke reconfiguration programme. In this article, we explore in detail how these decision-makers ‘held the line’ and ask what the broader power implications of doing so are for the interaction between evidence, health policy and system redesign.

**Methods:**

The research combined semi-structured interviews (n = 20) and documentary analysis of historically relevant policy papers and contemporary stroke reconfiguration documentation published by NHS London and other interested parties (n = 125). We applied a critical interpretive and reflexive approach to the analysis of the data.

**Results:**

We identified two forms of power which senior managerial decision-makers drew upon in order to ‘hold the line’. Firstly, discursive power, which through an emphasis on evidence, better patient outcomes, professional support and clinical credibility alongside a tightly managed consultation process, helped to set an agenda that was broadly receptive to the overall decision to change stroke services in the capital in a radical way. Secondly, once the essential parameters of the decision to change services had been agreed, senior managerial decision-makers ‘held the line’ through hierarchical New Public Management style power to minimise the traditional pressures to de-radicalise the reconfiguration through ‘top down’ decision-making.

**Conclusions:**

We problematise the concept of ‘holding the line’ and explore the power implications of such managerial approaches in the early phases of health service reconfiguration. We highlight the importance of evidence for senior managerial decision-makers in agenda setting and the limitations of clinical research findings in guiding politically sensitive policy decisions which impact upon regional healthcare systems.

## Background

Reconfiguration is “*a deliberately induced change of some significance in the distribution of medical, surgical, diagnostic and ancillary specialities that are available in each hospital or other secondary or tertiary acute care unit in locality, region or healthcare administrative area*” [1]. A growing international literature focuses on system wide health service reconfiguration, mergers and regional hospital service re-design in Europe [2–8] and North America [9–13]. These studies emphasise that reconfigurations are notoriously problematic and proposed solutions are frequently contested and bedevilled by conflict amongst different policy actors [1, 4, 12]. Furthermore, the subsequent results of new healthcare configurations are often perceived as underwhelming [7, 14]. In this article, we argue that a weakness in much of the existing reconfiguration literature is that the interplay between evidence, power and policy remains relatively uncharted. We propose to remedy this through an exploration of the reconfiguration of stroke services in London (2008–2012). This particular reconfiguration appears to have bucked the trend of most reconfigurations [7, 8, 15], because, despite the fact that some hospitals lost their stroke services altogether or saw them downgraded, it was implemented in full, on time, and overcoming both potential and real opposition from institutional actors and the wider public; this is unusual [7, 12, 14], marking the case as a ‘positive outlier’ [16]. The reformed London stroke service enjoyed broad political and popular support and has been advanced as a model of reference for other areas of England [17, 18]. Significantly, the new model is saving lives and is cost effective compared to other models of stroke care [19].

**Fig. 1:**
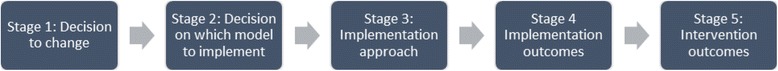
The case of the London reconfiguration, stages 1 and 2. Adapted from Fulop et al. [15]

Recent work by Fulop et al. [15] comparing stroke service reconfiguration in London and Manchester provides a valuable contribution to understanding system-wide reconfiguration. The authors identify five key stages in their analysis of the study of stroke service reconfigurations in London and Manchester and emphasise that, in the case of the London reconfiguration, stages 1 and 2 (Fig. 1) were “*led by the regional authority ‘holding the line’*” [15]. In this article, our focus is exclusively on these first two crucial stages. We explore how evidence was utilised and power mobilised by senior managers in order to ‘hold the line’ in the early stages of the reconfiguration. For us, ‘holding the line’ is a power-laden term, but interestingly, Fulop et al. [15] do not explicitly explore the role of power in their work. In linked research, Turner et al. [8] explore the role of ‘system leadership’ in designing the new model of stroke care provision in London, again in comparison to Manchester, and once more power is implicit but not explicit in their analysis.

We problematise this concept of ‘holding the line’ and ask what the broader power implications of ‘holding the line’ might be for evidence use, healthcare system redesign and the implementation of policy change. We draw on separate qualitative research conducted by a different research team with similar key informants to the Fulop et al. [15] and Turner et al. [8] work in London conducted at around the same time with a more explicit focus on power and the discourse of evidence based change. This research was funded separately from the work pursued by Fulop, Turner and their broader teams. However, both research teams were aware of each other’s work and discussed some early findings informally. We interpret power in post-structuralist terms [20–22], encouraging an analytical focus on language and discursive strategies to validate policy decisions. Discursive practice – who says what to whom (in speech or writing) in certain contexts, and the legitimacy derived from this use of language – may be seen to reflect, maintain or challenge power relations amongst different actors [22]. Nevertheless, discursive power sits alongside other forms of power (for example, hierarchical, managerial, jurisdictional and economic) in contemporary governance [21, 23, 24] and we also demonstrate how hierarchical power was utilised through managerial structures by the senior strategic level executive team leading the stroke care reforms in London.

We suggest that the discursive mobilisation of clinical research evidence to frame hospital reconfigurations in recent years is an increasingly important technique aimed at depoliticising contentious choices [4, 25], aligning with macro-level assertions around the importance of evidence-based medicine (EBM) to influence health policy decisions more generally [26–29] and that this was central to the London stroke service reconfiguration. Change framed as ‘evidence based’ has a rhetorical power that discourages dissent [25, 27] and a legitimating power that enables its proponents to establish jurisdiction over specific fields of practice [26, 30, 31]. This study draws on this existing literature and applies it to study how senior decision-makers mobilised the discourse of EBM as a technique of power [32, 33] so as to ‘hold the line’ [15] and provide ‘system leadership’ [8] in the critical early stages of the London stroke reconfiguration. We also show how senior strategic level managers harnessed hierarchical techniques of power to ensure their view of the newly configured services – how many specialist units, and where they would be located – was realised. We suggest health policy and systems research should unequivocally explore the concept of power and how it interacts with research evidence and managerial influence if we are to critically understand how complex reconfiguration policies are translated into practice [34–36].

## Methods

The research combined semi-structured interviews (n = 20) and documentary analysis of historically relevant policy papers and contemporary stroke reconfiguration documentation published by NHS London and in the public domain (n = 125). NHS London was the managerial body responsible for healthcare planning and delivery across the London region from 2006 to 2012. It was one of ten national Strategic Health Authorities (SHAs) in England. NHS London provided strategic supervision for the 31 Primary Care Trusts that commissioned local health services in London and also had oversight for some hospital services as well as ambulance and mental health services. We pursued a single case study approach that focused in detail and in depth on the processes and principles behind service delivery changes, namely what Stake [37] terms “*particularisation*”. Single case studies can demonstrate “*features or categories relevant to a wide number of settings*” [38]. This encourages ‘analytic’ rather than ‘enumerative induction’, focusing on transferability or generalisation in terms of theory rather than population [39].

We analysed two distinct types of documents. Firstly, the government commissioned strategic review of London Healthcare led by Darzi [40]. Official policy documents are useful for analysis because they seek to influence views, beliefs and actions [41]. We conducted a formal content analysis [42] of the Darzi review. The primary aim was to explore what motivates change, for example, economic drivers and/or service ‘quality’. The second documentary analysis focused on documentation published by NHS London as part of the public stroke consultation and implementation process building on the Darzi Review recommendations. These included stroke strategy documents and guidance to health service commissioners, NHS Trusts and other stakeholders. The analysis of these documents enabled us to explore how the discourse of evidence-based change was developed over time by NHS managers, management consultants and stroke professionals leading the London stroke reforms. Informed by analysis of the NHS London documents, we identified and approached key actors leading the reconfiguration for interview. The interviews focused on the pan-London governance changes of the reconfiguration of stroke services across London. The interview topic guide explored why and how stroke care was ‘problematised’ in London, the evidence behind the reconfiguration, managerial techniques to realise the goals of the reconfiguration, and potential impacts upon staff. At the heart of the governance arrangements for the stroke reconfiguration project in London sat a ‘Stroke Project Board’ (see Turner et al. [8], figure 1, p. 158 for a summary of the governance arrangements). The Stroke Project Board consisted of clinical and managerial actors, many of whom also sat on parallel ‘Clinical Expert’ and ‘Finance and Commissioning’ panels [43], which fed into the coordinating Stroke Project Board through the planning and development and consultation stages of the project.

Our sampling strategy aimed for depth rather than breadth of involvement, selecting informants who had served on the Stroke Project Board, Clinical Expert Panel or the Financing and Commissioning Panel. These individuals were purposively selected [44] because they were closely involved in key clinical and managerial decisions around the design and implementation of the reformed service and also involved in reviewing and recommending evidence to guide the reconfiguration. We interviewed eight predominantly ‘clinical’ informants (six senior stroke clinicians, representing medical, nursing and therapies viewpoints; one Public Health Professor; and one London Ambulance Service Assistant Medical Director), and 12 predominantly ‘managerial’ informants (five senior SHA managers, including Stroke Network Directors; two senior Stroke Project Managers; one Clinical Service Manager; two management consultants; and two stroke charity representatives). Sixteen of the informants were substantively employed by the NHS (in commissioning, management and/or patient facing roles) and four were not NHS employees. The non-NHS employees were particularly useful in independently gauging the ‘institutional’ influence of certain NHS actors; for example, as comparatively independent stakeholders, they were crucial to our ultimate analysis, especially where we found disagreement amongst some NHS informants. The fieldworker had established links with senior stroke and public health clinicians through work on a separate research project. Through these links he gained access to the wider circle of clinical and managerial informants. The fieldworker’s background in NHS management was also helpful in securing access to a number of informants.

The research was retrospective. The key strategic decisions behind the reconfiguration occurred over 2008–2009, whilst the interviews were conducted in 2011–2012. This historical approach is common in the social science literature relating to healthcare reconfiguration [5, 45]. Whilst recall can be a problem, there are advantages to a retrospective approach. A retrospective view on how and why processes developed as they did through the analysis of public documents combined with semi-structured key stakeholder interviews exploring drivers and objectives of change, enables critical examination of ‘stated and unstated drivers’ behind change [45]. NHS ethics applications were approved for the research.

All interviews were transcribed and analysed through Nvivo [46]. We applied a critical interpretive [47] and reflexive approach [48] to the analysis of the data. We explored how information is not only distributed, but produced, and how expertise, claims of truth and power dynamics can be interpreted and understood drawing on deductive analytic techniques producing primary codes linked to these themes [32, 33, 49]. The data analysis also generated other inductive themes. These could be functional (e.g. audit and standards, measurement, professional relations) or theoretical (e.g. conflict, specialisation). These inductive themes were drawn upon in the development of our two over-arching themes. For example, the theme of ‘specialisation’ helped our development of ideas linked to the ‘reification’ of stroke as an emergency condition requiring a new discursive approach and dynamic imagery. Likewise, audit, standardisation and improved measurement align closely with key facets of the New Public Management (NPM) [21].

The process then centred on building these fragments into larger theory – going between the data and the wider literature iteratively and exploring deviant cases [50]. Overall, we found great unanimity amongst our informants around the ultimate goals of the reconfiguration. Nevertheless, we identified some interprofessional and jurisdictional disputes amongst the accounts of some informants, particularly around the balance between rehabilitation and acute care reflected in the overall shape of the reconfiguration. We also identified tensions between institutional and ‘pan-London’ loyalties for clinical and managerial informants. These contributed to our analysis by encouraging us to consider the different ways in which evidence is mobilised by certain professional groups [51] and also how different actors identify with particular institutions.

## Results

Our results are structured to correspond with the first two stages of Fulop et al.’s [15] model (decision to change and decision on which model to implement; see Fig. 1 above) through which ‘holding the line’ was identified as significant. We explicitly analyse the concepts of evidence and power within these two stages.

### Decision to change

Senior SHA managers (who both set the remit for, and oversaw, the work of the central Stroke Project Board as introduced in the previous section) with wide ranging responsibility for pan-London healthcare skilfully harnessed the discursive power of ‘evidence’ from the very start of the stroke service reconfiguration process through voluminous documentation [40, 52, 53]. The Darzi Review of London health services, which introduced the concept of pan-London stroke reconfiguration, drew on the word ‘evidence’ 35 times in the 57,000-word text; an indicative extract from the introduction is cited below:

“*This report makes recommendations for change. It is based on a thorough, practitioner-led process, and rooted in evidence – gathered from a wide range of people and organisations from the world of healthcare and from the NHS’s partners in local government and beyond, from thorough reviews of the literature and data, and from the use of a range of analytical modelling techniques. It also reflects a major exercise to hear what Londoners say they want from their healthcare system.*” ([40], p. 4)

The collaborative, clinically led authorship of Darzi’s Report was heavily emphasised so that the appearance (at least) of how London’s stroke care inadequacies were ‘problematised’ [32, 33] became one of professional expertise rather than political or managerial decision-making. The perceptions of the problems that hindered effective stroke care and the prospective solutions were tightly controlled within a specialist, professional discourse [54]. There was no talk of financial rationalisation, or the need for mergers in stark contrast to earlier reviews of healthcare in London such as the Tomlinson Review of 1992 [55]. Rather, the Darzi Review emphasised the importance of clinical evidence and performance metrics (and implicitly, a form of rationality compatible to both professional and managerial stakeholders) in the pursuit of excellence for all – expressed by recurring phrases such as “*World Class Care*” [40]. The Darzi Review functions as a ‘blueprint’ for change with a useful degree of ‘strategic ambiguity’ [12] in that it enables clinical, managerial and public stakeholders from across London’s hospitals to agree on key principles of stroke care reform without pinpointing the details of where the reforms would take place, and thereby locate the institutional winners or losers.

The managerial team leading the stroke reconfiguration project (led by management consultants appointed by the SHA executive team) developed a communication strategy that explicitly advocated the use of clinicians:

“[A]*s ambassadors stressing the clinical basis for change’ and to ‘communicate* [the reconfiguration] *is clinically-led and will save lives*” ([56], p. 14)

In this way, it was not only the type of discourse: “*clinical… clinically led*” that was prescribed – but also the key spokespeople were selected to be those with clinical credibility and public trust in order to ‘hold the line’ as part of this communication strategy. Patient safety, and ‘life saving’ were prioritised in the framing of the reforms lending moral legitimacy to the programme [4, 25], alongside an economic rationale that highlighted the inefficiencies of prior practice [57, 58]. Having mobilised the discourse of evidence-based change, and ensured coherence of this message through clinically respected voices, senior managers also ensured that the parameters of any potential debates amongst public and professional stakeholders that might challenge the ‘line’ of the reforms were shaped by the project management team, as this informant acknowledges:

“[W]*e had our first pan London conference and that was attended by representatives across London… And we mixed everyone up and we eventually workshop*[ped ideas]*… But we kind of had the answers in our back pocket… And we were able to …* [guide] *that conference to an answer that we had already, I suppose, decided on…* [We] *were getting 150 to 200 people almost agreeing on 99.9% of what the problem was and how to solve it*.” (Management Consultant)

This quote gives a sense of how an agreed ‘line’ around the nature of the problem of stroke in the capital, and a way to resolving it, was managed by the project team during the first stages of the reconfiguration – indeed other informants spoke of the skill of management consultants in maintaining a coherent trajectory through these formative periods where stakeholder opinions were canvassed. Numerous informants highlighted the positive role played by ‘independent’ stroke charity stakeholders in publically backing the overall principles of the reconfiguration, i.e. fewer, more specialised stroke centres. Although the inclusive nature of the reconfiguration process was emphasised in official literature [40, 53], in reality, the options open to participants were limited as senior management figures effectively shaped how the need for the reforms was framed [25] and who was selected to make the case to the public, whilst carefully managing the discursive forums in which debate took place.

### Decision on which model to implement

In the previous section, we highlighted how the London stroke service reconfiguration was broadly framed as both evidence based and clinically led by senior SHA managers with overall responsibility for the reforms. We suggested that this was important in establishing both public and professional support for the reforms overall. In this section, we explore firstly the decision to select the particular ‘hub and spoke’ model of care chosen by senior SHA managers; secondly, we question how the decisions were made relating to which hospitals would be commissioned to provide specific services as part of the new model. The reformed London model is based around a ‘hub’ of eight Hyper Acute Stroke Units (HASUs), which exclusively admit all new stroke cases and provide specialist acute care for the first 72 hours post stroke onset. These eight HASUs are supported by 24 Stroke Unit ‘spokes’ that admit patients directly from HASU care once clinically indicated. It was a senior SHA management decision to implement an eight-site HASU model and to reject the 10- to 12-site HASU model as advised by the Clinical Expert Panel as part of the reconfiguration negotiations [8]:

“[M]*y attitude to it at the time was if we don’t dig our heels in and say it’s eight* [HASUs]*, I’m not interested at all unless it’s eight, it will end up being fifteen, it won’t end up being nine…* [I]*f we’re not careful, from that we’ll get a mushrooming of numbers, then we won’t have the results that we’re looking for. So the answer’s eight. So that was top down, one size fits all. And the reason for that was to prevent the traditional London solution of saying, ‘Well we’ll have both’*.” (SHA Senior Manager)

This decision represented a management judgement based on a determination to avoid the ‘inflationary’ [12] mistakes of other attempts at reconfiguration and to learn from historical failures in this area [8]. It also may reflect the fact that there was a paucity of evidence available at the time to demonstrate the appropriate size that a HASU should be with respect to activity levels. Rather than clinical research evidence guiding the choice of the eight-HASU model, it was in reality, a managerial calculation. The approach of the SHA was not consensual or deliberative around this question of site numbers, but firm and dictatorial [21]. This highlights the complementary importance of other – non-discursive – forms of power also at play in this reconfiguration [23]. The SHA executive enjoyed immense financial, hierarchical and goal-setting power in London at this time compared to the historical status quo ante in which strategic healthcare planning and direction in the capital tended to be much more fragmented [59–61]. The SHA executive team were able to draw on NPM style techniques (e.g. hands on, active, organisational control through explicit standards and performance measures [62]) to set targets for all the relevant NHS (both clinical and non-clinical) staff in London [21, 63] to mobilise action in line with their strategic goals linked to stroke improvement and hold a managerial ‘line’ emphasising the London-wide importance of stroke:

“*So your objectives for the year, every chief executive in London and every director here, I want to see where your contribution to taking these things forward figures in the top three things on your priorities… How are you, as a finance director, developing the stroke tariff in London? It should be at the top of your list just like you’re the chief nurse. What are you doing about the recruitment of specialist stroke nurses in London? … I insisted on those being at the top of everybody’s objectives. And therefore, when their appraisal was done, if they hadn’t done it, then they wouldn’t get a good grading*.” (SHA Senior Manager)

The hierarchical power of the SHA executive over key managerial and professional actors as demonstrated above highlights how senior managers could ‘hold the line’ throughout key NHS organisations and ensure that the goals of the executive were upheld with relation to the stroke reforms.

The decision about where to locate the eight proposed HASUs drew upon competing institutional considerations and negotiated priorities. London hospitals were invited to bid for accreditation to host one of the eight HASUs [64]. The SHA had devised a selection process that attempted to reconcile the potentially conflicting goals of service quality and patient access. The data showed that many found this a difficult undertaking:

“[I]*f you put quality criteria* [first]*, you get one group of eight* [hospital sites suitable for HASU care]*. If you put access criteria* [first]*, you get a different group. So making the best of those two was difficult and required adjustments to both*.” (SHA Senior Manager)

Our interview data highlight an example of how such ‘adjustments’ may have been made by senior SHA managers. Some informants indicated that, between the collation of the scores for individual hospital bids to provide HASU services being evaluated and the proposed reconfiguration being put out to public consultation, two ex post adjustments were made by the senior SHA executive team to the decision-making criteria. First, that HASU services ought to be co-located with specialised trauma services and, second, that HASUs ought to be co-located with specialist neuroscience services:

“[W]*e were asked* [by the SHA executive team] *to look at was there an evidence base which actually could be used to justify… the decision that those places that had major trauma also had to have a hyper acute unit in? And actually I think the answer was frankly, ‘no’…* [T]*here are no interdependencies really between hyper acute stroke care and major trauma care.*” (SHA Stroke Project Manager 1)

“[W]*e put together an argument as to why you had to have trauma and stroke together. It took a bit of time because you kept on having to say ‘Well there is no argument.’…we’d produce the piece of paper as to why it had to be together…* ***If someone had said they’d got to be separate, we could have forced it through as well*** *.*” (Emphasis added) (SHA Stroke Project Manager 2)

“[It was] *like a rabbit out of a hat and it really was that startling – and I recollected being at the meeting in which decisions were taken. The message was delivered from on high, by which I took to be* [senior SHA managers]*, that there has to be co-location, which significantly advantaged one of the hospitals and disadvantaged another.*” (Stroke Charity representative)

In this instance then, for these informants, ‘holding the line’ equated to the development of ex post criteria by the SHA executive team and an instruction to project managers that they search for evidence to justify co-location. Whilst the project managers were asked to engage in the discursive practice of “*put*[ting] *an argument together*” and producing documentation to suggest that there was an evidence base to justify these decisions, the hierarchical power of senior figures within the SHA is highlighted by the view expressed by the second project manager above that whatever the executive team had requested: “*we could have forced it through as well*”. Some informants suggested in interviews that the impacts of these decisions left them feeling rather alienated as the result of these decisions presaged the downgrading of one of the best performing stroke services in London, thus opening the HASU location process up to accusations of ‘goalpost shifting’ amongst some of the members of the Stroke Project Board and the shaping of evidence to fit policy rather than the other way around:

“[T]*he big controversy at the time was around HASUs, and actually it had gone from being a really transparent, really robust process that everyone believed in, to being a bit make-it-up-as-you-go-along and find evidence where you need it because it justifies your political end basically. So it suddenly went from being very transparent to being very suspicious*.” (Senior Clinician)

As highlighted in the quote above, whilst the senior SHA managers alienated some stakeholders over this issue – crucially – this did not derail or delay the overall programme of reform. Ultimately, the ‘line was held’ during this second stage of the reconfiguration [15]. There are a number of reasons why this was the case. Firstly, not all stakeholders were necessarily aware of the significance of the co-location ‘adjustment’ in the HASU site location decision-making process, and it is certainly the case that no such wider public awareness of the ‘controversy’, as highlighted above, permeated further than individual members of the decision-making team. Secondly, our interview data highlight that a ‘pan-London ethos’, or London stroke specialist clinical identity emerged amongst those involved in designing the new city-wide services, which ultimately trumped traditional institutional loyalty. Thirdly, some senior clinicians whose local services were downgraded were appointed to senior pan-London stroke leadership roles:

“*So when he was appointed to the job, before we confirmed the job, I saw him and said, ‘If you’re going to do this job, you’ve got to not, you’ve got to forget about keeping* [a HASU service at your own hospital]*,’ and he’d come round to the view that the right thing to do was take forward the eight* [HASU model]*… Then it’s fantastic, because then if anyone wants to say, ‘Well I don’t like this because I’m losing my singlehanded neurologist from Nether Wallop DGH,’ wherever it is, he says, ‘You want to share your pain with me, my friend? I’ve just had to preside over the closure of* [a very high performing] *unit in London, so what’s your problem?*” (SHA Senior Manager)

The willingness of such senior clinicians to promote the overall reforms in spite of the impacts these had on their own institutions added to the overall credibility of the model and was useful for minimising further clinical dissent and thus ‘holding the line’ [15]. The implications of these results are now discussed.

## Discussion

In this article, we showed how the SHA executive management team reflectively developed and operationalised a clinical, evidence-based discourse to frame problems and identify solutions building on the work of the Darzi Review into London’s health services. The dual ethical and economic rationale behind the proposed changes [65] was emphasised in documentation aimed at the public and professionals, and a communications strategy [56], which identified clinicians as key messengers and called for them to be directed to highlight the life-saving nature of the reforms, was established [4, 25]. In this respect, the goals of the senior SHA managers and stroke specialist clinicians were aligned in the first stage of the reconfiguration process – decision to change [15, 66]. However, when it came to the second stage of the reconfiguration process – decision on which model to implement [15], given the lack of clinical evidence to guide how many HASUs would be optimal, and the difficulty of reconciling quality and access criteria to guide exactly where they ought to be located, we presented data to show that the SHA executive management team utilised ‘top down’ [21] managerial strategies to limit the centralised HASU model to eight sites and devised what were perceived by some on the Stroke Project Board as ex post ‘adjustments’ to justify decisions about HASU site location. In this respect, the goals of the senior SHA managers and some stroke specialist clinicians diverged, as this led to an existing high performing stroke unit effectively being downgraded. Notwithstanding this controversy, the reconfiguration proceeded and the overall ‘line was held’.

The practical implications of this study are linked to the fact that the London stroke reforms have in recent years become a model of reference for ‘successful reconfiguration’ [7, 17, 18]. Whilst the clinical results may be impressive [19], it is important that we appreciate the messy and contested trade-offs that shaped this high profile service reconfiguration and explore the forms of power mobilised by senior SHA executives at different times and contexts in order to ‘hold the line’. The reconfiguration process was characterised by strong leadership from the SHA executive team [8, 15] and effective presentational control over key strategic messages. The discursive ‘line was held’ through an effective framing and communications strategy led by the SHA, as was the managerial ‘line’, through goal alignment and target setting for key figures within the connected NHS organisations accountable to the SHA in London. Interview data collected from key actors involved in the reconfiguration suggest that some (but not all) informants perceived that important elements of the decision-making criteria around HASU location were adjusted ex post and that this impacted upon the robustness and transparency of the overall process. In practical terms, this paper seeks to illuminate hitherto hidden perspectives around how such complex and contested decisions and the mobilisation of managerial power to maintain strategic coherence may be experienced by individual stakeholders.

In theoretical terms this work highlights the importance of the discourse of EBM as a technique of power in that it establishes new standards, or norms, through which problems are constructed and understood [28, 33]. This is helpful in understanding the broad unanimity of purpose amongst clinicians, managers, public and other stakeholders with respect to the depoliticised framing of the pan-London stroke reforms and gaining legitimacy for the decision to change services in principle [15]. With respect to the decision on which model to implement in practice [15], however, the bounded and contested nature of the actual evidence available and the difficulty of aligning the competing values of service quality and patient access, illustrates the limits of the discourse of EBM and the importance of other forms of power that enable managers to pursue their strategic goals [21]. It is therefore important to consider the interaction of discursive power and hierarchical forms of managerial power and their implications for implementation [35] and health systems research and policy more broadly [21, 34, 36].

A limitation of our study is the lack of public and patient participation. Whilst we focus on how research evidence was utilised and power mobilised by senior decision-makers in order to ‘hold the line’ in the early stages of the reconfiguration, it would be useful to explore public and patient understandings of these phenomena [7] both in the United Kingdom and internationally.

## Conclusion

We suggest that this article offers important empirical and theoretical contributions. In empirical terms, we identified the key processes and techniques through which senior decision-makers set the agenda around the decision to change, and the decision on which model to implement in the London stroke care reforms of 2008–2012. We harnessed documentary and interview data to add a new dimension to the overall understanding of this high profile and highly regarded reconfiguration. In theoretical terms, we critically explored the concept of ‘holding the line’ and problematised [32, 33] this in relation to how research evidence was mobilised and power was exerted by senior SHA managers to secure desired outcomes in this complex clinical service reconfiguration. We identified two forms of power that the SHA executive team drew upon in order to ‘hold the line’; firstly, discursive power [20–23], which through an emphasis on clinical evidence, better patient outcomes, professional support and clinical credibility, alongside a tightly managed consultation process, helped to shape a (public, political and institutional) context that was broadly receptive to the overall decision to change stroke services in the capital in a radical way. Secondly, once the essential parameters of the decision to change services had been agreed, the SHA executive team ‘held the line’ through hierarchical NPM style power [21, 63] to minimise traditional ‘inflationary’ pressures to de-radicalise reconfiguration plans [12] through ‘top down’ decision-making. We conclude that it is important to explicitly consider the interplay between research evidence, power and policy in studies of health service reconfiguration in order to get a deeper understanding of the roles played by different actors in setting agendas and shaping new systems. In the new post-SHA NHS landscape, the implications of these findings may be of use to those tasked with delivering, designing and evaluating the Sustainability and Transformation Plans in England [67], as well as those exploring service redesign elsewhere in the United Kingdom and beyond [68].
